# Bio-inspired electronic fingerprint PUF device with single-walled carbon nanotube network surface mediated by M13 bacteriophage template

**DOI:** 10.1038/s41598-022-24658-9

**Published:** 2022-11-22

**Authors:** Jae-Seung Jeong, Gyo Sub Lee, Tae-Eon Park, Ki-Young Lee, Hyunsu Ju

**Affiliations:** 1 Korea Institute of Science and Technology, 5, Hwarang-ro 14-gil, Seongbuk-gu, Seoul, 02792 Republic of Korea; 2 ASML Korea, 25, Samsung 1-ro 5-gil, Hwaseong-si, Gyeonggi-do Republic of Korea; 3 University of Science and Technology of Korea (UST), 217, Gajeong-ro, Yuseong-gu, Daejeon, 34113 Republic of Korea

**Keywords:** Engineering, Materials science, Nanoscience and technology

## Abstract

Human fingerprints are randomly created during fetal activity in the womb, resulting in unique and physically irreproducible fingerprint patterns that are applicable as a biological cryptographic primitive. Similarly, stochastically knitted single-walled carbon nanotube (SWNT) network surfaces exhibit inherently random and unique electrical characteristics that can be exploited as a physical unclonable function (PUF) in the authentication. In this study, filamentous M13 bacteriophages are used as a biological gluing template to create a random SWNT network surface with mechanical flexibility, with electrical properties determined by random variation during fabrication. The resistance profile between two adjacent electrodes was mapped for these M13-mediated SWNT network surfaces, with the results demonstrating a unique resistance profile for each M13-SWNT device, similar to that of human fingerprints. Randomness and uniqueness measures were evaluated as respectively 50.5% and 50% using generated challenge–response pairs. Min-entropy for unpredictability evaluation of the M13-SWNT based PUFs resulted in 0.98. Our results showed that M13-SWNT random network exhibits cryptographic characteristics when used in a bio-inspired PUF device.

## Introduction

The semiconductor industry has offered various security systems, but traditional security methodologies still face tremendous threats from sophisticated attacks^1–3^. To protect against these attacks, physical unclonable functions (PUFs) employ the inherent physical characteristics of electrical devices arising from fabrication variations^4^. These characteristics vary randomly and are unduplicable, as they result from inherent structural variations. Diverse PUF implementations have been proposed based on optical devices, RFID, FPGA, integrated circuits, memory devices, organic electronics, and carbon nanotubes (CNTs)^5–14^. CNT-based PUFs have attracted significant attention for wearable applications owing to their flexibility and printable characteristic^11,12^. Prior studies have been conducted on the implementation of CNT-based PUFs^1,14^. These PUFs exploit an analog resistance network using a random conducting path formed by dispersed CNTs during the fabrication^10^. This analog resistance network has desirable inherent randomness for PUF fabrication. Moreover, CNT-based PUFs also have sufficient tolerance to ultraviolet light and radiation, increasing their reliability in various fields^1^. In this study, M13 phages were employed as a biological glue layer to apply single-walled carbon nanotubes (SWNTs) to a M13 nanomesh^13,15^ and implement a unique CNT-based PUF device (M13-SWNT). The SWNTs provide large effective surface areas, forming percolating structures, and provide efficient interfacing with ionic systems (electrochemical, biological, and biochemical), mechanical flexibility, and optical transparency characteristics^13^. The M13 phage, as a biological glue material, is strongly bound to the SWNTs to assemble the conductive nanomesh^15^. Consequently, the M13-SWNT surface arrangement is formed randomly from inevitable fabrication variations, and this inherent feature enables PUF device implementation in cryptographic key generation.

## Materials and methods

### Device characteristics

**Figure 1:**
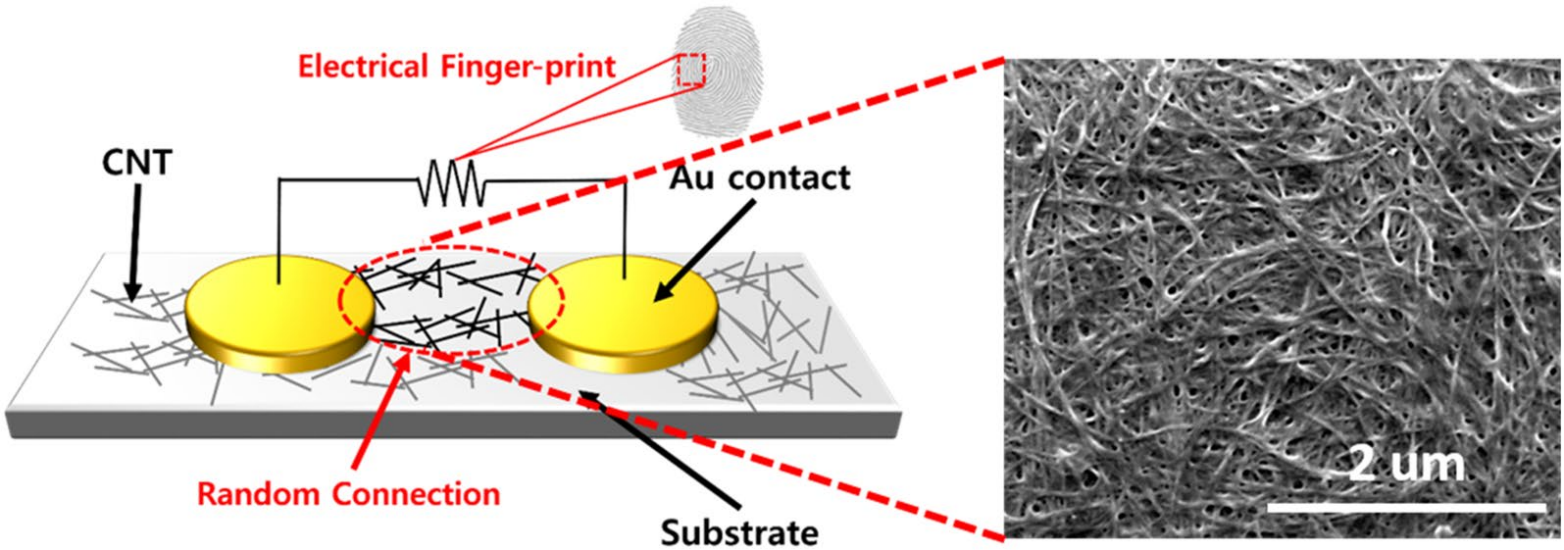
Schematic of M13-SWNT-based PUF. The random resistance values are attributed to the variable M13-SWNT network surfaces, which cause the unpredictable responses.

Fabrication variation makes it physically impossible to duplicate security chips and, therefore, is essential for PUF device implementation. The M13-SWNT film was fabricated through hydrodynamic assembly process as previous published^13^. The SWNT (purchased from Nanointegris Inc.) has the diameter varying from 1.2 to 1.7 nm with the mean diameter of 1.4 nm and the length distributed from 100 nm to 4 um with the average ~ 1 um. The hydrodynamic assembly method used for M13-SWNT-based PUF devices has been reported to increase fabrication variation. Briefly, SWNTs dispersed in a sodium cholate solution were mixed with a genetically engineered M13 bacteriophage. This M13 bacteriophage was used because of its filamentous nature and strong binding affinity for SWNTs, resulting from a specific peptide sequence on its body surface (p8 peptide). A mixture of SWNTs and an M13 bacteriophage solution, based on a 4:1 molar ratio of SWNT to M13 bacteriophage, was dialyzed against DI water. Through the hydrodynamic assembly method, M13 works as a biological glue to facilitate the large area of SWNT film^13^. An M13-SWNT-conductive network film formed around the inner wall of the dialysis membrane via a hydrodynamic assembly process because of concentration polarization. Because this phenomenon is not exactly the same in the entire area, M13-SWNT film has a locally different resistance. These random networks apparently formed as a result of non-reproducible variables such as wrinkles and curvature of the membrane. Au dot contacts of 10 × 10 were deposited by thermal evaporation and the diameter of the Au contact was 300 um with the center-to-center distance of 650 um. During the fabrication a randomly distributed surface is developed on the M13-SWNT substrate, forming resistance networks between the two electrodes (Fig. 1). M13-SWNT-based PUF devices fabricated in the same batch exhibit unique resistance networks. Similar to human fingerprints, these resistance values produce characteristic electric response patterns for the M13-SWNT-devices.

**Figure 2:**
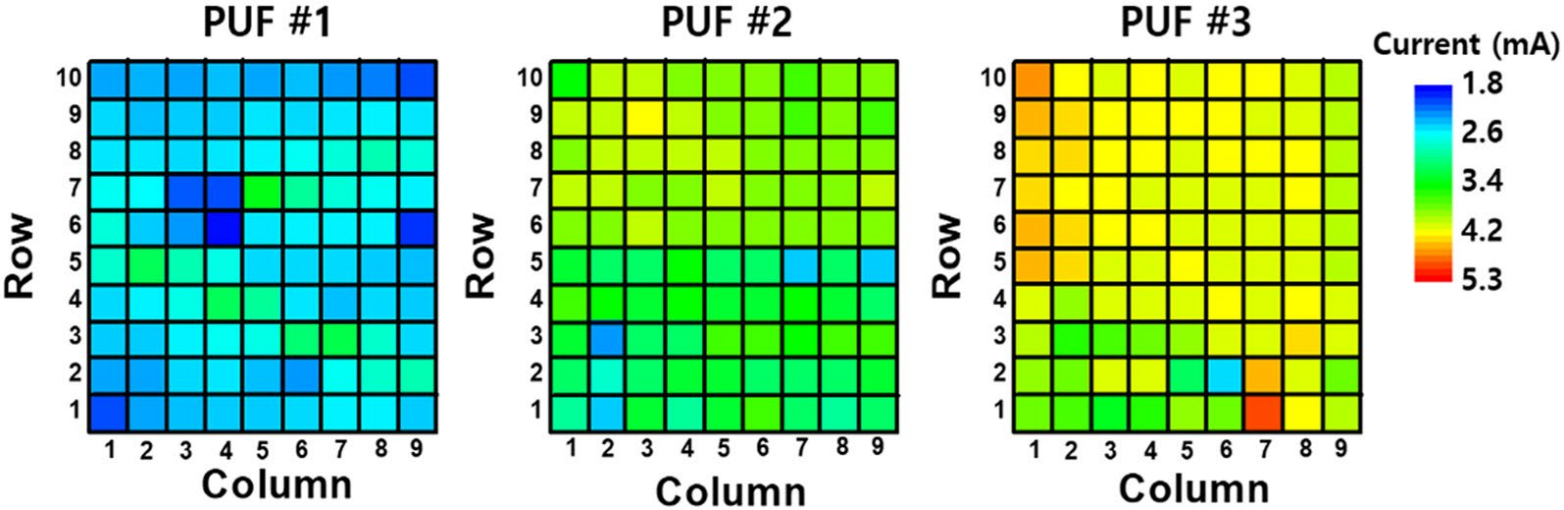
Resistance variation of carbon nanotube-based conductive nanomesh. Each pixel indicates the cell current value measured for 0.5 V/10 ns. All M13-SWNT based PUFs have different resistance values at the same position, indicating that they can generate different responses to the same challenges.

Resistance distributions were investigated by applying a voltage pulse of 0.5 V/100 ns to measure the read current of 90 cells for three different M13-SWNT-based PUFs fabricated in the same batch. Figure 2 represents the variations in the measured resistance. The resistance values of the M13-SWNT-based PUFs exhibit random patterns due to cell-to-cell variation, which indicates the likeliness of duplicating these PUFs. The following section (results and discussion) will analyze these random patterns using quantitative methods. Moreover, the significant resistance variation in the M13-SWNT material can improve authentication characteristics.

### Challenge–response pair (CRP) generation method

**Figure 3:**
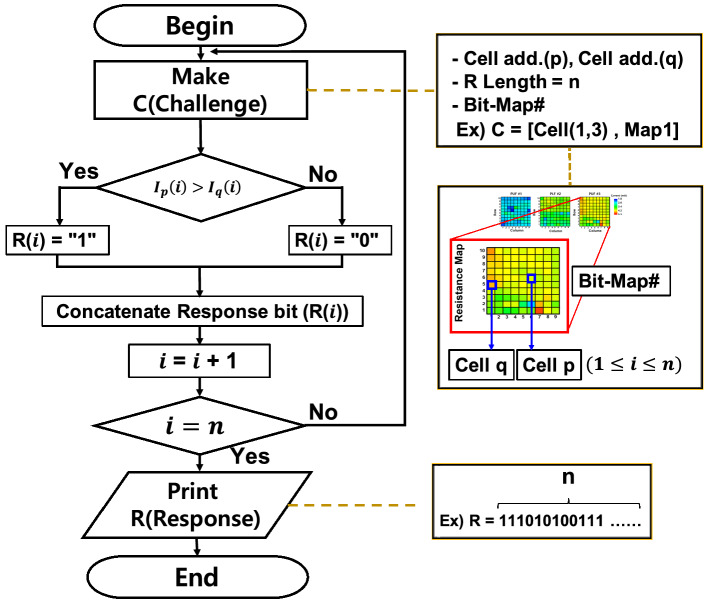
Schematic of CRP generation algorithm. When a request for CRP generation arrives, a challenge (C) is generated that comprises two particular cells from a specific PUF device and a response length (R). Comparing the currents of the first and second selected cells produces a bit represented by 0 or 1. Concatenating each bit into the response bits, the CRP is completed as a response of $n$ bits.

The PUF, as a cryptographic primitive, should respond to paired challenges with prearranged outputs, together referred to as challenge–response pairs (CRP). The response bit, 0 or 1, is generated by comparing the currents of the two cells selected by the challenge (Fig. 3). This CRP generation method predicts the response-bit generation mechanism more difficult than using CRP generation with a predefined reference cell because the possible cell combinations become much larger. Furthermore, it may help the response bits avoid a bias to 0 or 1, even when the current values of the cells follow a biased distribution within a specific window. Thus, the total possible combinations are $$\left( {\begin{array}{*{20}c} {\text{m}} \\ 2 \\ \end{array} } \right)^{{\text{n}}}$$, where m is the number of cells in a PUF device, and n is the bit length of the response string.

## Results and Discussion

### Min-entropy

Min-entropy is the most conservative way to measure the unpredictability of a set of outcomes and is evaluated by the responses as follows^16^:

1 $${\text{H}}_{{{\text{min}}}} = - \log_{2} \left( {{\text{P}}_{{{\text{max}}}} } \right),$$

where ${\text{H}}_{{{\text{min}}}}$ denotes the min-entropy of the samples, and ${\text{P}}_{{{\text{max}}}}$ maximum probability of 0 or 1 at each position of the response to the challenges.

2 $$\left( {{\text{H}}_{{{\text{min}}}} } \right)_{{{\varvec{total}}}} = - \frac{1}{{\varvec{n}}}\mathop \sum \limits_{{{\varvec{i}} = 1}}^{{\varvec{n}}} \log_{2} \left( {{\text{P}}_{{{\text{max}}}} } \right)$$

If *P*_max_ is close to 0.5, then the min-entropy leads to an ideal value of 1. The response patterns from the PUF with a min-entropy close to 1 become almost unpredictable. All the fabricated M13-SWNT-based PUFs had a desirably high min-entropy of 0.98, regardless of the individual PUF cell distribution, demonstrating the unpredictability of their responses.

### Randomness and uniqueness evaluation

**Figure 4:**
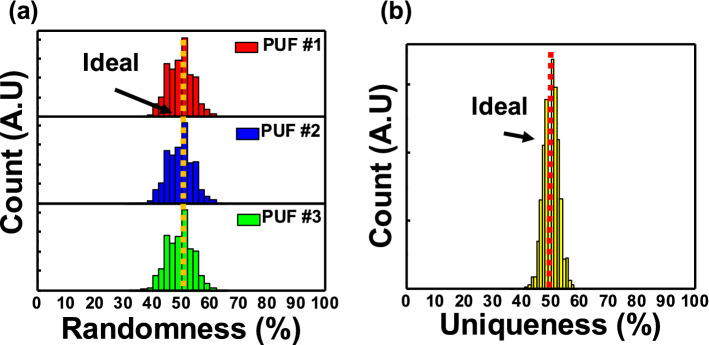
Randomness and uniqueness of the M13-SWNT based PUFs. (**a**) The randomness of each M13-SWNT based PUFs fabricated in the same batch is close to the ideal value of 50%. (**b**) The uniqueness of M13-SWNT based PUFs close to the ideal value of 50%.

Randomness evaluates the unpredictability of the responses and is obtained by measuring the number of ‘1 s’ or ‘0 s’ in the response string^17^. An ideal PUF should have randomness of 50%, which contributes to strong tolerance against brute-force attacks. Uniqueness represents how different responses are expected to be when the same challenge is applied to different PUFs^17^. It is evaluated by measuring the hamming-distance between responses of different PUFs to the same challenge, and an ideal PUF should have a uniqueness of 50%. The randomness was measured by applying 10,000 different challenges and extracting the 240-bit responses from each PUF. The uniqueness was also evaluated by applying the same challenges 10,000 times to the three PUFs and obtaining the 240-bit responses. The randomness of the M13-SWNT-based PUFs results was 50%, 50.5%, and 51%, all of which are close to the ideal value of 50%, as shown in Fig. 4a. Moreover, the uniqueness of PUFs also tended to the ideal value of 50%, as shown in Fig. 4b.

### Environment variations

**Figure 5:**
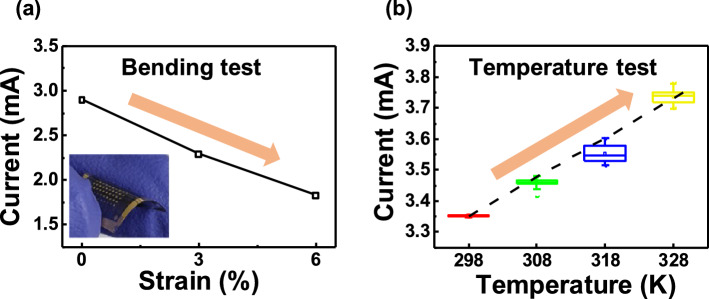
Resistance variations of the M13-SWNT based PUFs caused by environmental variations. (**a**) Explicit negative relation between the current and the bending strain on the substrate and (**b**) positive tendency of the current to the temperature variation.

The PUF device is required to behave reliably by reproducing the same responses even under environmental variations. In particular, the M13-SWNT-based PUFs with a flexible substrate are easily exposed to physical and temperature variations, and these changes often cause a bit flip in the response electrical outputs. However, when the electrical changes can be linearly correlated with environmental variation, the corresponding relationship between resistance and environmental variation can be used to minimize the possibility of bit flips, in a process referred to as error correction^18^. Therefore, our study investigated the dependencies of resistance on bending and temperature variation. When the M13-SWNT-based PUF was subjected to bending, resistance increased, with respect to strain, (Fig. 5a). Moreover, a temperature increase from 25 to 50 °C linearly decreased the resistance, indicated by increased current flow shown (Fig. 5b). Based on the linear correlation of resistance with these environmental variables the bit errors induced by environmental change can be suppressed via a compensation algorithm.

## Conclusion

A single-walled carbon nanotube (SWNT) network surface was implemented for a PUF application using a M13 bacteriophage layer as a biological glue material through a simple hydrodynamic assembly process. This process can naturally form random SWNT connections between the two electrodes through inherent fabrication variations. The random connection variations lead to a random and unique resistance distribution for each M13-SWNT device. Individual cells in the M13-SWNT device were defined by the resistance between two adjacent electrodes. These randomly distributed and unique resistance values were then used to generate the challenge–response pairs (CRPs) for a cryptographic primitive. To evaluate the M13-SWNT-based PUF devices, the randomness, uniqueness, and min-entropy were determined for the given CRPs. In addition, resistance was found to have a linear correlation with environmentally induced temperature and strain changes. These relationships can be used to compensate for resistance change, and thus minimize the bit errors. We successfully demonstrated the cryptographic properties of M13-SWNT and its robustness to environmental variation when used as a biomimetic PUF device.

## Data Availability

The datasets generated and/or analyzed during the current study are not publicly available due to follows the intellectual-property protection guidelines in KIST as a national research institute, but are available from the corresponding author on reasonable request.
